# Modulation of Cell Proliferation by Macrophages: A Possible Function Apart from Cytotoxic Tumour Rejection

**DOI:** 10.1038/bjc.1974.214

**Published:** 1974-11

**Authors:** R. Keller

## Abstract

**Images:**


*Br. J. Cancer* (1974) **30**, 401

### MODULATION OF CELL PROLIFERATION BY MACROPHAGES: A POSSIBLE FUNCTION APART FROM CYTOTOXIC TUMOUR REJECTION

R. KELLER

*From the Immunobiology Research Group, University of Zurich, Schönleinstrasse 22, CH-8032 Zurich/Switzerland*

Received 21 June 1974. Accepted 16 July 1974

**Summary.—The *in vitro* interaction between activated, non-immune macrophages (AM) and a variety of syngeneic, allogeneic or xenogeneic " normal " and " malignant " target cell lines was followed by different parameters such as target cell proliferation, viability or morphology.**

**Proliferation of all rapidly replicating cell lines examined, irrespective of whether they were of syngeneic, allogeneic or xenogeneic origin, or showed normal or neoplastic growth characteristics, was similarly blocked by the presence of AM in an effector/target cell ratio of 10 : 1. It was only in very slowly proliferating cells that this inhibitory effect was not detectable. A marked diminution in target cell proliferation was also achieved with target cells growing in suspension, where maintenance of close contact between effectors and targets is unlikely, indicating that this macrophage effect may be mediated by a soluble product of AM.**

**The finding of clear differences in the proliferation inhibition of slowly proliferating normal and neoplastic targets suggested that proliferation *per se* may not fully mirror the consequences of the macrophage/target cell interaction. This was affirmed when viability and morphology were used as parameters: viability was virtually unaffected in normal targets whereas neoplastic cells were killed.**

**Accordingly, it is suggested that activated non-immune macrophages can affect targets in strikingly different ways. Inhibition of proliferation could be an important homoeostatic regulatory function of the macrophage which would affect every replicating cell. Cytocidal killing of targets, on the other hand, is achieved only on neoplastic cells.**

RECENT studies in many laboratories have led to the recognition that macrophages can contribute in a variety of ways to host resistance against tumours. The long established immunologically specific cytotoxic tumour cell killing (Granger and Weiser, 1964) is achieved not only by macrophages sensitized with specific antibody but also by normal macrophages which have come in contact with a soluble product of sensitized lymphocytes plus specific antigen (Evans and Alexander, 1972). Moreover, in cultures of sensitized macrophages and specific antigens, antigenically unrelated tumour cells can be damaged as " innocent bystanders " (Evans and Alexander, 1972). Finally, normal macrophages activated *in vivo* and *in vitro* by any of a large array of nonspecific stimulants of natural immunity acquire the capacity effectively to inhibit tumour growth *in vitro* (Alexander and Evans, 1971; Keller and Jones, 1971; Keller, 1973*a*, *b*; Hibbs, Lambert and Remington, 1972; Holtermann, Klein and Casale, 1973) and *in vivo* (Keller and Hess, 1972).

Nonspecific killing of tumour targets has generally been viewed as cytotoxic in nature. However, our own investigations have suggested that such target cell killing may be a good deal more than a single step reaction and probably involves a mechanism which initially expresses itself by a marked decrease in target cell proliferation (Keller, 1973*a*).* In the present study, the *in vitro* interaction between activated, non-immune macrophages (AM) and a variety of syngeneic, allogeneic and xenogeneic " neoplastic " and " normal " cell lines was examined by cell proliferation, residual cell numbers and morphological changes as a means of following the manner and magnitude of effector/target cell interaction. The findings reported here show that in the presence of an appropriate ratio of AM, target cell proliferation is always markedly reduced or completely stopped in all quickly replicating cell lines, " normal " as well as " neoplastic ". However, other generally employed parameters of interaction, such as morphology and viability, were greatly altered in neoplastic but not in targets derived from normal tissues. Thus, the rapid growth of " normal " cells is slowed or stopped by AM, whereas transformed or tumour cells are quickly rendered *non-viable* by an as yet unidentified cytocidal process.

### MATERIALS AND METHODS

*Animals.*—Inbred rats of the DA strain were used throughout; inbred Lewis rats were used in a few experiments. Animals were maintained under conventional conditions. The pathogen-free DA rats used in one experiment were kindly supplied by the Institut für Biologisch-Medizinische Forschung AG, Füllinsdorf/Switzerland.

*Cell lines.*—DA rat tumours were the ones previously described (Keller, 1973*a*) or were newly induced by the injection of polyoma virus or dimethylbenzanthracene (DMBA), or by instillation of methylcholanthrene (MCA; Keller, 1973*a*). An MCA tumour has also been induced in inbred Lewis rats. These cells were grown in Eagle's minimal essential medium (MEM; Eagle, 1959) modified as follows: glutamine 280 mg/l, calcium 100 mg/l, $NaHCO_3$ 1 g/l, glucose 2 g/l and biotin 1 mg/l, and supplemented with penicillin 100 *E*/ml, streptomycin 50 $\mu$g/ml (modified MEM) and 10% newborn calf serum (NCS). DA and Lewis adult (from the diaphragm) and embryonic fibroblast cultures were established after tryptic digestion of tissues and grown in RPMI 1640 medium supplemented with 20% foetal calf serum (FCS). Cells derived from normal rat kidney (NRK) and rat kidney cells infected with $B_{77}$-Rous sarcoma virus ($B_{77}$-NRK; Duc-Nguyen, Rosenblum and Zeigel, 1966), a gift from Dr H. P. Schnebli, were grown in modified MEM supplemented with 10% FCS and 10% tryptose phosphate (Difco).

Balb/c Simian virus 40-($SV_{40}$) transformed 3T3 cells and Balb/c 3T3 cells were a gift from Dr Stuart Aaronson and were maintained in modified MEM with 10% NCS.

CHO hamster fibroblasts, a gift from Dr W. Schmid, were grown in modified MEM supplemented with 10% NCS.

A human fibroblast line (Lopez), a gift from Dr W. Schmid, was grown in modified MEM containing 10% FCS. A cell line derived from a human mammary carcinoma (BT 20) was a gift from Dr Ch. Sauter, and was cultivated in RPMI 1640 medium supplemented with antibiotics and 2% FCS. Three human melanomata were kindly provided by Dr K. T. Brunner. SK-melanoma-1 was grown in suspension in RPMI 1640 supplemented with 10% NCS. Melanoma-13 and melanoma 4-2 were grown in adherent culture in RPMI 1640 supplemented with 20% FCS.

*Preparation of macrophage monolayers.*—Peritoneal cells were obtained from DA rats 3 days after intraperitoneal injection of 10 ml of 10% peptone (Fluka AG, Buchs SG) by washing out the cavity with RPMI 1640 medium. Cultures of rat peritoneal macrophages were prepared by seeding approximately $2 \times 10^6$ macrophages into 35 × 10 mm Falcon plastic Petri dishes (Keller, 1973*a*); when larger numbers of effector and/or target cells were cultured, or when inter-

* In the present work, inhibition of target cell proliferation without significant signs of cytotoxicity (release of $^{51}Cr$, uptake of trypan blue) is viewed as a purely cytostatic effect; interactions accompanied by an increase in $^{51}Cr$ release and in uptake of trypan blue, and usually a loss of viability of targets were termed cytotoxic; in interactions followed by a marked *decrease* in the number of targets without any evident parallel increase in cytotoxicity, it is assumed that targets are *killed* by a presently unknown cytocidal mechanism.

action was followed over a period of several days, 60 × 10 mm Falcon plastic Petri dishes were used. The macrophages were allowed to adhere for 30 min at 37°C. The non-adhering cells were then removed by intensive washing of the monolayers with jets of tissue culture fluid. After this procedure, at least 96% of the cells remaining on the dishes showed the characteristics of macrophages (morphology, adherence and phagocytosis). These cells were designated activated macrophages (AM). Target cells (usually $2 \times 10^5$ cells/dish) were then immediately added to the monolayer. If not stated otherwise, the effector/target cell ratio was 10 : 1. Cultures were in RPMI 1640 medium supplemented with 10% NCS, usually maintained at 37°C in a humid atmosphere of 5% $CO_2$/95% air.

*Assessment of effector/target cell interaction.*—To assess the kind and degree of effector/target cell interaction, the morphology of the cultures was followed at varying intervals by phase contrast microscopy or after fixation with methanol and staining with Giemsa.

Cytotoxicity was assessed in some of the cell lines labelled with $^{51}Cr$ (sodium chromate; Eidgen. Institut für Reaktorforschung, Würenlingen, Switzerland; 50 $\mu$Ci $^{51}Cr/10^8$ cells) using the methods described previously (Keller, 1973*a*), or by determining the percentage of cells which had taken up trypan blue.

Residual target cell proliferation was measured after varying intervals of effector/target cell interaction by exposure for 60 min at 37°C to 1 $\mu$Ci [$^3$H]-thymidine/dish ([$^3$H]-TdR; methyl-[$^3$H]; 5000 mCi/mmol; The Radiochemical Centre, Amersham, Buckinghamshire, England) and washed 3 times with 1·5% perchloric acid. To ensure that the radioactive precursor was incorporated into DNA, nucleic acid radioactivity was measured in a liquid scintillation counter after it had been solubilized with hot perchloric acid (Keller, 1973*a*). Data are reported as disintegrations/min (d/min).

In cloning experiments target cells were harvested by trypsinization of 3-day old monolayers. After washing, $2 \times 10^5$ cells were cultured in the presence of $2 \times 10^6$ macrophages in RPMI 1640 medium supplemented with 10% NCS. After 72 h, the cells were harvested by trypsinization, washed and counted, and when necessary diluted to a concentration of 300 target cells per 3·5 ml; aliquots of this volume were dispensed to 30 ml Falcon culture flasks. After 10 days' incubation in 5% $CO_2$ at 37°C, target cell proliferation was assessed by addition of 1 $\mu$Ci [$^3$H]-TdR for 60 min at 37°C and processing the cells as described.

### RESULTS

Some investigators have given the impression that the well documented effect of AM on tumour targets might be operational only in unusual situations in which macrophage activation occurs secondary to another event such as infection with facultative intracellular organisms. Experiments in pathogen-free rats were made to determine whether this distinctive effect on targets was peculiar to the presence of host microbial pathogens or whether it was inherent to normal macrophages.

Results represented in Table I show

TABLE I.—*Similarity in Inhibition of Cell Proliferation by Macrophages from Conventional and Pathogen-free Rats**

| | Macrophages from conventional rats | | Macrophages from pathogen-free rats | |
|---|---|---|---|---|
| | Residual proliferation (% of control) after interaction for (h) | | | |
| Target cells | 4 | 12 | 4 | 12 |
| Polyoma induced DA rat tumour | 47 (±4·8) | 1 (±0) | 13 (±2·5) | 0·3 (±0·5) |
| Hamster fibroblasts CHO | 40 (±5·0) | 1 (±0) | 4 (±1·6) | 0·6 (±1·1) |
| Human fibroblasts | 23 (±2·7) | 1 (±0·5) | 8 (±1·9) | 1 (±0) |

* Effector/target cell ratio was 10 : 1
Each value represents the mean of 16 determinations.

that macrophages taken from pathogen-free rats 3 days after intraperitoneal injection of 10% peptone profoundly inhibited the proliferation of target cells. Accordingly, macrophage cytostatic potential would seem to be an inherent property of these cells and can be raised to a highly potent level by the very same means known to lead to macrophage activation.

*The presence of activated macrophages on target cell proliferation* in vitro

The derivation and the growth characteristics of the various cell lines used in this study are listed in Table II. Studies on these revealed that the proliferation of these lines is affected differently by the presence of AM. Results with representative categories of target cells, *i.e.* malignant *vs* normal syngeneic, allogeneic *vs* xenogeneic, are depicted in Fig. 1. These experiments demonstrate most dramatically that proliferation of rapidly proliferating syngeneic rat tumour cell lines is totally blocked after culture for 24–48 h with AM at an effector/target cell ratio of 10 : 1. This finding applies to two different polyoma induced tumours, maintained in culture over 80 (represented in Fig. 1) and 4 passages respectively, and for two syngeneic carcinogen induced tumours. Proliferation of methylcholanthrene induced tumour cells from a Lewis rat which differs from the DA strain in major histocompatibility antigens (Palm, 1971) was similarly inhibited by AM from DA rats, as were syngeneic cell lines. The proliferation of other malignant rat ($B_{77}$), mouse ($SV_{40}$ 3T3) and human (BT 20; melanoma 4-2) cell lines growing in adherent culture and of human SK-melanoma-1-cells growing in suspension, was blocked in a manner comparable with syngeneic cell lines.

Proliferation of lines derived from normal cells was affected differently by AM. The incorporation of tritiated thymidine by fibroblasts derived from embryonic (Fig. 1) or from adult syngeneic DA rat tissues was essentially unaffected as long as their proliferation rate remained low (Table III). After a few passages, however, when their proliferation rate had increased, the extent of the macrophage cytostatic effect became essentially comparable with that exerted on malignant cell lines (Table III). Proliferation of allogeneic embryonic fibroblasts from Lewis rats was similarly affected by AM, as was proliferation of syngeneic fibroblasts; *i.e.*, hardly any effect was seen on early passage line, but marked effects were evident on the later passage cells. With other lines derived from normal

TABLE II.—*Origin and Growth Characteristics of the Cell Lines Employed*

| Cell line | Origin | Number of passages *in vitro* | Proliferation (range in d/min × 100) 4 h | 72 h |
|---|---|---|---|---|
| Polyoma I | DA rat | 70–80 | 5·5–139 | 73–1390 |
| Polyoma II | DA rat | 3–7 | 5·6–178 | 22–1600 |
| Methylcholanthrene | DA rat | 30–36 | 5·3–168 | 50–1600 |
| Dimethylbenzanthracene | DA rat | 2–4 | 5–147 | 28–1700 |
| Methylcholanthrene | Lewis rat | 10–17 | 4–148 | 197–1750 |
| Normal rat kidney | rat | ∞ | 6·4–24 | 31–400 |
| $B_{77}$ Rous sarcoma virus | rat | ∞ | 7·8–17 | 43–88 |
| Embryonic rat fibroblasts | DA rat | 0–3 | 0·79–1·4 | 2·6–12·7 |
| | | 6–12 | 9·4–15 | 78–122·5 |
| Adult rat fibroblasts | DA rat | 0–3 | 0·7–2·5 | 2·6–8·9 |
| Embryonic rat fibroblasts | Lewis rat | 0–3 | 0·7–1·65 | 10·5–31·5 |
| 3T3 | Balb/c mouse | ∞ | 7–72 | 25–1600 |
| $SV_{40}$ 3T3 | Balb/c mouse | ∞ | 9–145 | 60–1600 |
| CHO | hamster fibroblasts | ∞ | 6·7–69 | 17–775 |
| Lopez | human fibroblasts | ∞ | 7–142 | 12–1700 |
| BT 20 | human adenocarcinoma | ∞ | 7·5–17 | 21·2–27 |
| SK-mel-1 | human melanoma (suspens.) | ∞ | 12–110 | 650–843 |

∞ *in vitro* culture was for years.

FIG. 1.—The effect of the presence of $2 \times 10^6$ DA rat macrophages on the proliferation of $2 \times 10^5$ target cells. Target cell lines: ■ slowly proliferating embryonic DA rat fibroblasts; ● human fibroblasts; ▲ polyoma induced DA rat tumour cells.

TABLE III.—*Correlation between Macrophage Cytostasis and the Proliferation Rate of Syngeneic Rat Embryonic Fibroblasts*

| Target passage | Proliferative capacity (range in d/min) | | Residual target cell proliferation * (% of control) after culture for various intervals with AM (effector/target cell ratio = 10 : 1) — Duration of interaction with AM of DA strain (h) | | | | | | |
|---|---|---|---|---|---|---|---|---|---|
| | 4 h | 72 h | 4 | 12 | 24 | 36 | 48 | 60 | 72 |
| 0–2 | 79–140 | 260 1270 | 94·6 | 82·3 | 90·8 | 86·3 | 103 | 57·0 | 65·3 |
| 6–12 | 940–1500 | 7800–12250 | 25·0 | 19·0 | 5·0 | 2·5 | 1·2 | 4·0 | 7·5 |

* For the proliferation range of the cell lines see Table II.
Each value represents the mean of 10 determinations.

tissues and proliferating at a rather high rate (normal rat kidney, mouse 3T3, hamster fibroblast CHO, human fibroblasts Lopez; Table II), the presence of macrophages consistently produced a marked inhibition of proliferation (represented by Lopez fibroblasts in Fig. 1, and Table I). The data are thus indicative of the macrophage cytostatic effect being largely correlated with the proliferation rate or with cell membrane phenomena that parallel it; in any case it is neither species-specific nor tumour-specific. However, the observation that in slowly proliferating human adenocarcinoma cells (BT 20; Table II) thymidine incorporation

TABLE IV.—*Contrast in Cytostasis on Slowly Proliferating Malignant* vs. *Slowly Proliferating Normal Cells*

| Type of target | Residual target cell proliferation * (% of control) after culture with AM in ratio of 10 AM : 1 target — Duration of interaction with AM (h) 4 | 12 | 24 | 36 | 48 | 60 | 72 |
|---|---|---|---|---|---|---|---|
| Adult DA rat fibroblasts | 90 (±5) | 126 (±34) | 96 (±1) | 72 (±10) | 65 (±6) | 87 (±2) | 82 (±4) |
| Human adenocarcinoma BT 20 | 20 (±14) | 10 (±2) | 5 (±2) | 8 (±6) | 3 (±1) | 2 (±1) | 4 (±6) |

* For the proliferation range of the cell lines see Table II.
Each value represents the mean of 12 determinations.

is much more inhibited by AM than incorporation by slowly proliferating embryonic fibroblasts suggests that quite apart from their rate of replication (Table IV) there may be still other significant differences in the macrophage reaction against normal and malignant cells.

*The presence of activated macrophages on target cell viability*

Differences in the macrophage cytostatic effect upon slowly proliferating normal and slowly proliferating malignant cell lines and the observation of marked cytostasis on rapidly proliferating normal and transformed cells do not by themselves resolve the crucial question whether this effect is irreversible. Accordingly, other parameters of effector/target cell interaction relating directly to target cell viability were followed. These criteria included release of $^{51}Cr$, uptake of trypan blue, the residual number of target cells in the cultures, and their cloning efficacy. In harmony with earlier observations, signs of cytotoxicity such as employed in lymphocyte target studies, release of $^{51}Cr$ or uptake of trypan blue, remained within the same range, irrespective of whether targets were cultured alone or in the presence of AM (Table V). It is noteworthy that despite the lack of the aforementioned criteria of cytotoxicity, of phagocytosis or of the presence of cell debris, parallel morphological observations revealed a progressive, striking decrease in the number of tumour targets.

Although the number of targets remaining in the dishes following culture with AM could not always be assessed accurately because removal of cells by trypsinization made differentiation between effectors and targets sometimes difficult the direct comparison with control cultures at the end of the 72 h incubation period in most cases led to clear conclusions. Data such as that given in Table VI revealed striking differences between the cell lines examined. In accordance with the results of proliferation studies, both syngeneic (DA) and allogeneic (Lewis) rat fibroblasts grown in the presence of AM were found to be only slightly decreased in number or not at all. Although proliferation of non-transformed mouse (3T3) and human (Lopez) fibroblasts (cf. Fig. 1) were almost completely blocked by macrophages, the actual number of these targets was hardly diminished (Table VI). In marked contrast, the number of syngeneic (polyoma) and xenogeneic ($SV_{40}$ 3T3) tumour cells that remained after interaction with AM was consistently drastically reduced; indeed, targets were often completely eliminated. The observations showing that there is a progressive disappearance of tumour targets in the absence of injured cells or of cell debris is indicative of the involvement of a special, cytocidal process.

In other experiments, targets grown for 72 h in the presence of AM were cloned and thymidine incorporation was assessed after a further 10 days of culture. Results in Table VII show that malignant cell lines exposed to AM for 72 h were no

TABLE V.—*Failure of "Standard" Cytotoxicity Tests to Disclose Effects on Target Cells Exerted by AM*

| | Target cells cultured with AM* | | | | | | | | | |
|---|---|---|---|---|---|---|---|---|---|---|
| | Polyoma | | $SV_{40}$ 3T3 | | 3T3 | | ERF | | ARF | |
| Cytotoxicity tests | Control | +AM | Control | +AM | Control | +AM | Control | +AM | Control | +AM |
| $^{51}Cr$ release (%) ++ | 50 (±7) | 56 (±8) | 50 (±2) | 24 (±2) | 32 (±2) | 41 (±5) | 46 (±7) | 29 (±6) | 35 (±2) | 31 (±6) |
| Uptake of trypan blue (% positive cells)† | 1 (±0·3) | 1 (±0·5) | 1 (±0·3) | 2 (±0·8) | 1 (±0·2) | 2 (±0·4) | 0·2 (±0·3) | 1 (±0·6) | 1 (±0·3) | 1 (±0·4) |

Each value represents the mean of 15 determinations.

* The number of targets was $2 \times 10^5$, the number of AM $2 \times 10^6$ per dish.

++ Incubation was for 12 h.

† Incubation was for 48 h.

Target cells: Polyoma: Polyoma induced cells from DA rat strain

$SV_{40}$ 3T3: $SV_{40}$-transformed Balb/c 3T3 cells

3T3: Balb/c 3T3 fibroblasts

ERF: Embryonic DA rat fibroblasts

ARF: Adult DA rat fibroblasts.

TABLE VI.—*Activated Macrophages are Selectively Cytocidal for Tumour Targets*

| Cultures | Approximate no. of surviving targets present in culture after 72 h | | | | | |
|---|---|---|---|---|---|---|
| | Polyoma | $SV_{40}$ | 3T3 | Lopez | ERF-DA | ERF-Lewis |
| $2\times10^5$ targets, alone | $2–5\times10^6$ | $2–5\times10^6$ | $3–6\times10^5$ | $5\times10^5–1\cdot4\times10^6$ | $2\cdot2–5\cdot6\times10^5$ | $2\cdot3–2\cdot7\times10^5$ |
| Targets$+2\times10^6$ AM | $0–6\times10^2$ | $0–6\times10^2$ | $1\cdot1–1\cdot7\times10^5$ | $1\cdot3–1\cdot7\times10^5$ | $1\cdot5–2\times10^5$ | $1\cdot4–1\cdot7\times10^5$ |

Each value represents the mean of 6 experiments.

Target cells: Polyoma: Polyoma induced tumour cells from DA rat strain

$SV_{40}$: $SV_{40}$ transformed Balb/c 3T3 cells

3T3: Balb/c 3T3 fibroblasts

Lopez: Human fibroblasts

ERF-DA: Embryonic DA rat fibroblasts

ERF-Lewis: Embryonic Lewis rat fibroblasts.

TABLE VII.—*Loss of Cloning Capacity of Tumour Targets after Interaction with AM: Validation of AM Cytocidal Effects as Being Selective*

| Target cell line | Syngeneic polyoma induced | Mouse $SV_{40}$ 3T3 | Mouse 3T3 | Syngeneic rat fibroblasts | Allogeneic rat fibroblasts | Human fibroblasts | Hamster fibroblasts |
|---|---|---|---|---|---|---|---|
| Cloning efficacy* (% of control; ±S.D.) | 1 | 4 | 79 | 93 | 108 | 76 | 55 |
| | (±2) | (±5) | (±9) | (±17) | (±21) | (±14) | (±7) |

* Each value represents the mean of 6–10 determinations.

longer able to re-establish growth; morphological examination confirmed the incorporation data. On the other hand, culture with AM of syngeneic or xenogeneic cell lines derived from normal tissues diminished their cloning efficacy only slightly. These data thus confirm the prior estimations of residual target cell numbers following interaction with AM and thus demonstrate that viability of normal and neoplastic targets is affected differently by AM. The reduced cloning efficiency of CHO hamster fibroblasts, a cell line carried through countless passages, might reflect some more subtle kinds of changes recognized by macrophages.

*Morphological aspects of interaction between AM and various target cell lines*

The observation that only in transformed cells was the macrophage cytostatic effect paralleled by a corresponding decrease in target cell viability, as opposed to cell lines derived from normal tissues, pointed to a rather subtle but important difference in the way by which AM interact with normal compared with transformed target cells. To obtain further information on the character of this critical distinction, the morphological consequences of the interaction between AM and target were explored over a 72 h period. Signs of close cell-to-cell contact, resulting in occasional large aggregates of targets and effectors, were most pronounced in polyoma induced syngeneic and allogeneic rat tumours, less marked in carcinogen and Rous sarcoma virus induced rat tumours, not clearly detectable in some other neoplastic lines ($SV_{40}$ 3T3; BT 20) and were absent in all normal cell lines examined. Morphological target cell alterations such as shrinking were most obvious in syngeneic and allogeneic rat tumours, and in $SV_{40}$ transformed mouse fibroblasts. Although a decrease in the number of targets occurred in all these tumour situations, this was especially marked in virus induced rat and mouse tumours (Fig. 2a, b). In the carcinogen induced syngeneic and allogeneic rat tumours and the slowly proliferating human mammary carcinoma cells, these consequences of interaction were somewhat less pronounced but were nonetheless consistently evident (Fig. 3a, b). In both virus and carcinogen induced rat tumours, the morphological consequences of the interaction with AM have been followed on more than 100 separate occasions; Fig. 2 and 3 are representative of the alterations consistently evident after 72 h culture. As mentioned previously, the number of targets was consistently markedly decreased and in some preparations totally absent although neither signs of phagocytosis nor cell debris were detected. Cells derived from normal tissues, although mostly inhibited in proliferation as a consequence of their interaction with AM, at no time showed these morphological alterations (Fig. 4a, b).

### DISCUSSION

The *in vitro* interaction between activated macrophages and a spectrum of targets has been examined, including various normal and transformed syngeneic, allogeneic or xenogeneic cell lines and a number of parameters to evaluate proliferation, viability and morphology as meaningful consequences of interaction. A number of new findings and rather unexpected data have emerged. The prior observation (Keller, 1973*a*; 1974) that tumour cell proliferation is quickly and markedly decreased by AM was confirmed and extended. The cytostatic macrophage effect proved to be similarly potent against syngeneic, allogeneic or xenogeneic tumour targets whether autochthonous or induced by viruses or by carcinogens. Morever, proliferation of rapidly replicating cell lines derived from normal tissues of varied origin was blocked almost as rapidly and completely as that of neoplastic targets. However, the observed marked difference in the degree of the cytostatic macrophage effect upon slowly

FIG. 2a.—Polyoma induced DA rat tumour cells grown for 72 h. Phase contrast microscopy. ×125.

FIG. 2b.—Polyoma induced DA rat tumour cells derived from the very same culture and grown under identical conditions as indicated in Fig. 2a, but in the presence of $2 \times 10^6$ activated, non-immune DA rat peritoneal macrophages. In the whole preparation, only a few shrunken tumour cells were still present. Phase contrast microscopy. ×125.

FIG. 3a.—Methylcholanthrene induced DA rat tumour cells grown for 72 h. Phase contrast microscopy. ×125.

FIG. 3b.—Methylcholanthrene induced DA rat tumour cells derived from the same actual culture and grown under identical conditions as indicated in Fig. 3a, but in the presence of $2 \times 10^6$ DA rat AM. Tumour cells were reduced in number and the remaining cells were shrunken. Phase contrast microscopy. ×125.

FIG. 4a.—Adult DA rat fibroblasts grown for 72 h. Phase contrast microscopy. ×125.

FIG. 4b.—Adult DA rat fibroblasts derived from the same actual culture and grown under identical conditions as indicated in Fig. 4a, but in the presence of $2 \times 10^6$ DA rat AM. Although the number of fibroblasts is slightly decreased, their morphology is similar to controls. Phase contrast microscopy. ×125.

proliferating normal fibroblasts and slowly proliferating tumour cells (Table VI) revealed what may prove to be an important distinction. It now seems unlikely that the rapid and marked cytostatic effect on human tumour cells is due solely to the rate at which these cells proliferate; this rate is only slightly higher than that of rat fibroblasts. This issue cannot be resolved conclusively until observations can be made on a number of slowly proliferating tumour targets (not presently available to us) or, more likely, until something of the molecular mechanisms involved in the macrophage cytostatic effect becomes known.

The present experiments thus attest to the inherent capacity of macrophages to block the proliferation of any rapidly dividing cells. This effect seems quite independent of tumour, cell, or species specificity; proliferation of rapidly replicating normal cells and transformed cells is affected comparably. This AM effect can thus be differentiated from that of known inhibitors of cell proliferation such as interferon (Gresser and Bourali, 1970) or the chalones (Houck, 1973; Bullough, 1973). Further observations (Keller, 1973*a*; Waldman and Gottlieb, 1973) suggest to us that the potent homoeostatic capacity of macrophages to limit cell proliferation may be modulated depending on the ratio of effectors and targets. This could be interpreted as indicative of another intriguing host homoeostatic means for regulating cell proliferation.

The data on targets derived from normal tissues and on targets grown in suspension argue against the prior notion that close contact between effectors and targets is essential for blocking of cell proliferation. In suspension culture of rapidly proliferating melanoma cells, close contact with AM could not have occurred consistently since targets were non-adherent, in contrast to previously studied monolayer tumour targets. Despite the absence of aggregates of targets and AM as morphological evidence of close contact, the cytostatic process proved fully efficient. Our earlier view (Keller, 1973*a*) based on experiments with syngeneic polyoma and carcinogen induced tumour cells that close cell-to-cell contact and blockade of cell proliferation were causally related is thus no longer tenable. The present findings would be more consistent with this action of macrophages being mediated by a soluble factor elaborated by these cells. Data in support of this interpretation have been obtained and will be reported separately.

How is it that a cystotatic effect of AM on normal cells of such magnitude and uniformity had not previously been seen by the investigators in this field? In retrospect, this is more readily understandable as the omission relates primarily to the methods used to assess the consequences of effector-to-target cell interaction. So far, conclusions on the outcome of such interactions have been based solely on the morphological consequences, *i.e.* either a decrease in number and changes in shape of targets as observed in the cultures (Hibbs *et al.*, 1972; Hibbs, 1973) or enumeration of targets remaining detectable after interaction (Evans and Alexander, 1972; Alexander and Evans, 1971; Holtermann *et al.*, 1973). Using these parameters, cytocidal, cytolytic or cytotoxic effects accompanied by a marked diminishment in tumour target numbers were accurately reflected without in any way disclosing, however, of the less obvious cytostatic effects. In this respect, work showing that macrophages can effectively modulate the responses of lymphocytes to a variety of stimuli provides information and clues for such effects on targets (Perkins and Makinodan, 1965; Parkhouse and Dutton, 1966; Diener, Shortman and Russell, 1970; Yoshinaga, Yoshinaga and Waksman, 1972; Scott, 1972*a*, *b*; Sjöberg, 1972). These observations have demonstrated that antibody formation by lymphoid cells or their *in vitro* responsiveness to phytohaemagglutinin, endotoxin or allogeneic lymphocytes was inhibited by the presence of macrophages. More recent work indicates (Waldman and Gottlieb, 1973; Keller, (unpublished); Nelson, 1973) that regulation of DNA synthesis in lymphoid cells by macrophages is mediated by a soluble factor.

For a better understanding of the effector/target cell interaction, other parameters such as target cell viability and morphology were followed. The present data on the release of $^{51}$Cr from, and the uptake of trypan blue by targets following interaction with AM provided no evidence for a major role of classic cytotoxicity in the present system and are thus in keeping with previous observations (Keller, 1973*a*). However, in view of the fact that but few selected target cell lines have the qualities (including sensitivities) to warrant their use in assays of lymphocyte mediated cytotoxicity these criteria obviously have serious limitations; thus, a conclusive decision on this aspect of the macrophage effect would be premature.

Morphological observations made early in the course of our work in this field showed that tumour targets were markedly diminished in number in the presence of AM (Keller and Jones, 1971; Keller, 1973*a*; 1974) have been confirmed and many more such observations were made in the present work. Such drastic reduction in target cell number was detected in every neoplastic cell line examined. In contrast, studies of cell lines derived from normal tissues showed that in most lines the final number of targets was not at all affected by the presence of AM; in one xenogeneic line there was a moderate reduction in cell number. In this respect, the differences appearing in the morphological consequences of the macrophage interaction with normal or tumour targets were similar to those observed in various other *in vitro* systems (Hibbs *et al.*, 1972; Holtermann *et al.*, 1973; Hibbs, 1973). These morphological observations were confirmed by enumerating the number of targets still present after 72 h interaction with AM (Table VI). In an even more objective way, results of cloning experiments have shown that neoplastic targets cultured for 72 h with AM were quite incapable of re-establishing growth, whereas cells derived from normal tissues were consistently able to resume growth. Although the array of tumours examined could be more extensive, the present data may suffice to attest that tumour targets are selectively and effectively killed during their *in vitro* culture with AM. The repeated observations of a progressive decrease in the number of tumour cells without detectable signs of classic cytotoxicity, of phagocytosis or of cell debris, all suggest that the striking potency of tumour target elimination by AM is due to an as yet undefined cytocidal process.

The present data, taken as a whole, show that AM interacting with targets derived from normal tissues stop target cell proliferation without substantially affecting viability. In sharp contrast, interaction of AM with neoplastic cells not only blocks their replication but subsequently kills these targets by a cytocidal process. These macrophage effects on cell proliferation are nonspecific in the sense that there appear to be no species, cell type or tumour limitations in this action and involve all cell types examined, even including mitogen activated lymphocytes (Waldman and Gottlieb, 1973; Keller (unpublished); Nelson, 1973). In this respect, macrophages could qualify for an important role in host homoeostatic regulation of cell proliferation. Control of mitotic cell division, a universal and fundamental aspect of all eukaryote life is in some respects the keystone or epicentre of the cancer problem; such a regulatory mechanism might well play a central role in host resistance against malignant disease. The present evidence supports an even more basic role of the macrophage in cell proliferation generally, and provides a further example of the subtlety of the interrelations between different types of cells.

I am grateful to Dr R. Wyler, Institute of Virology, University of Zurich, for his help in producing polyoma induced rat tumours and for the assessment of possible mycoplasma contamination of the cultures; I thank Dr S. Aaronson, National Cancer Institute, NIH, Bethesda, Md. USA; Dr T. Brunner, Institut Suisse des Recherches expérimentales sur le Cancer, Lausanne, Switzerland; Dr Ch. Sauter, Medizinische Universitätsklinik, Kantonsspital Zürich; Dr W. Schmid, Kinderspital Zürich, and Dr H. P. Schnebli, Friedrich Miescher-Institut, Basel, Switzerland, for their generous gift of cell lines, and Dr Maurice Landy, Schweizerisches Forschungsinstitut, Davos, Switzerland, for his helpful criticism of this manuscript. The expert technical assistance of Miss R. Keist and Miss E. Bösch is gratefully acknowledged. This work was supported by the Swiss National Science Foundation (grant No. 3.516.71).

### REFERENCES

ALEXANDER, P. & EVANS, R. (1971) Endotoxin and Double-stranded RNA Render Macrophages Cytotoxic. *Nature, New Biol.*, **232**, 76.

BULLOUGH, W. S. (1973) The Chalones: a Review. *Natn. Cancer Inst. Monog.*, **38**, 5.

DIENER, E., SHORTMAN, K. & RUSSELL, P. (1970) Induction of Immunity and Tolerance *in vitro* in the Absence of Phagocytic Cells. *Nature, Lond*, **225**, 731.

DUC-NGUYEN, H., ROSENBLUM, E. N. & ZEIGEL, R. F. (1966) Persistent Infection of a Rat Kidney Cell Line with Rauscher Murine Leukemia Virus. *J. Bact.*, **92**, 1133.

EAGLE, H. (1959) Amino Acid Metabolism in Mammalian Cell Culture. *Science, N.Y.*, **130**, 432.

EVANS, R. & ALEXANDER, P. (1972) Mechanism of Immunologically Specific Killing of Tumour Cells by Macrophages. *Nature, Lond.*, **236**, 168.

GRANGER, G. A. & WEISER, R. S. (1964) Homograft Target Cells: Specific Destruction *in vitro* by Contact Interaction with Immune Macrophages. *Science, N.Y.*, **145**, 1427.

GRESSER, I. & BOURALI, C. (1970) Antitumour Effects of Interferon Preparations. *J. natn. Cancer Inst.*, **45**, 365.

HIBBS, J. B. (1973) Macrophage Nonimmunologic Recognition: Target Cell Factors Related to Contact Inhibition. *Science, N.Y.*, **180**, 868.

HIBBS, J. B., LAMBERT, L. H. & REMINGTON, J. S. (1972) Macrophage Mediated Non-specific Cytotoxicity—Possible Role in Tumour Resistance. *Nature, New Biol.*, **235**, 48.

HOLTERMANN, O. A., KLEIN, E. & CASALE, G. P. (1973) Selective Cytotoxicity of Peritoneal Leucocytes for Neoplastic Cells. *Cell Immun.*, **9**, 339.

HOUCK, J. C. (1973) General Introduction to the Chalone Concept. *Natn. Cancer Inst. Monog.*, **38**, 1.

KELLER, R. (1973*a*) Cytostatic Elimination of Syngeneic Rat Tumour Cells *in vitro* by Non-specifically activated Macrophages. *J. exp. Med.*, **138**, 625.

KELLER, R. (1973*b*) Evidence for Compromise of Tumour Immunity in Rats by a Non-specific Blocking Serum Factor that Inactivates Macrophages. *Br. J. exp. Path.*, **54**, 298.

KELLER, R. (1974) Mechanisms by which Activated Normal Macrophages Destroy Syngeneic Rat Tumour Cells *in vitro*. Cytokinetics, Non-involvement of T Lymphocytes, and Effect of Metabolic Inhibitors. *Immunology* **27**, 285.

KELLER, R. & HESS, M. W. (1972) Tumour Growth and Nonspecific Immunity in Rats: the Mechanisms Involved in Inhibition of Tumour Growth. *Br. J. exp. Path.*, **53**, 570.

KELLER, R. & JONES, V. E. (1971) Role of Activated Macrophages and Antibody in Inhibition and Enhancement of Tumour Growth in Rats. *Lancet*, *ii*, 847.

NELSON, D. S. (1973) Production by Stimulated Macrophages of Factors Depressing Lymphocyte Transformation. *Nature, Lond.*, **246**, 306.

PALM, J. (1971) Immunogenetic Analysis of AG-B Histocompatibility Antigens in Rats. *Transplantation*, **11**, 175.

PARKHOUSE, R. M. E. & DUTTON, R. W. (1966) Inhibition of Spleen DNA Synthesis by Autologous Macrophages. *J. Immun.*, **97**, 663.

PERKINS, E. H. & MAKINODAN, T. (1965) The Suppressive Role of Mouse Peritoneal Phagocytes in Agglutinin Response. *J. Immun.*, **94**, 765.

SCOTT, M. T. (1972*a*) Biological Effects of the Adjuvant *Corynebacterium parvum*. I. Inhibition of PHA, Mixed Lymphocyte and GVH Reactivity. *Cell Immun.*, **5**, 459.

SCOTT, M. T. (1972*b*) Biological Effects of the Adjuvant *Corynebacterium parvum*. II. Evidence for Macrophage-T-cell Interaction. *Cell Immun.*, **5**, 469.

SJÖBERG, O. (1972) Effect of Allogeneic Cell Interaction on the Primary Immune Response *in vitro*. Cell Types Involved in Suppression and Stimulation of Antibody Synthesis. *Clin. & exp. Immunol.*, **12**, 365.

WALDMAN, S. R. & GOTTLIEB, A. A. (1973) Macrophage Regulation of DNA Synthesis in Lymphoid Cells: Effects of a Soluble Factor from Macrophages. *Cell Immun.*, **9**, 142.

YOSHINAGA, M., YOSHINAGA, A. & WAKSMAN, B. H. (1972) Regulation of Lymphocyte Response *in vitro*. I. Regulatory Effect of Macrophages and Thymus-dependent (T) Cells on the Response of Thymus-independent (B) Lymphocytes to Endotoxin. *J. exp. Med.*, **136**, 956.


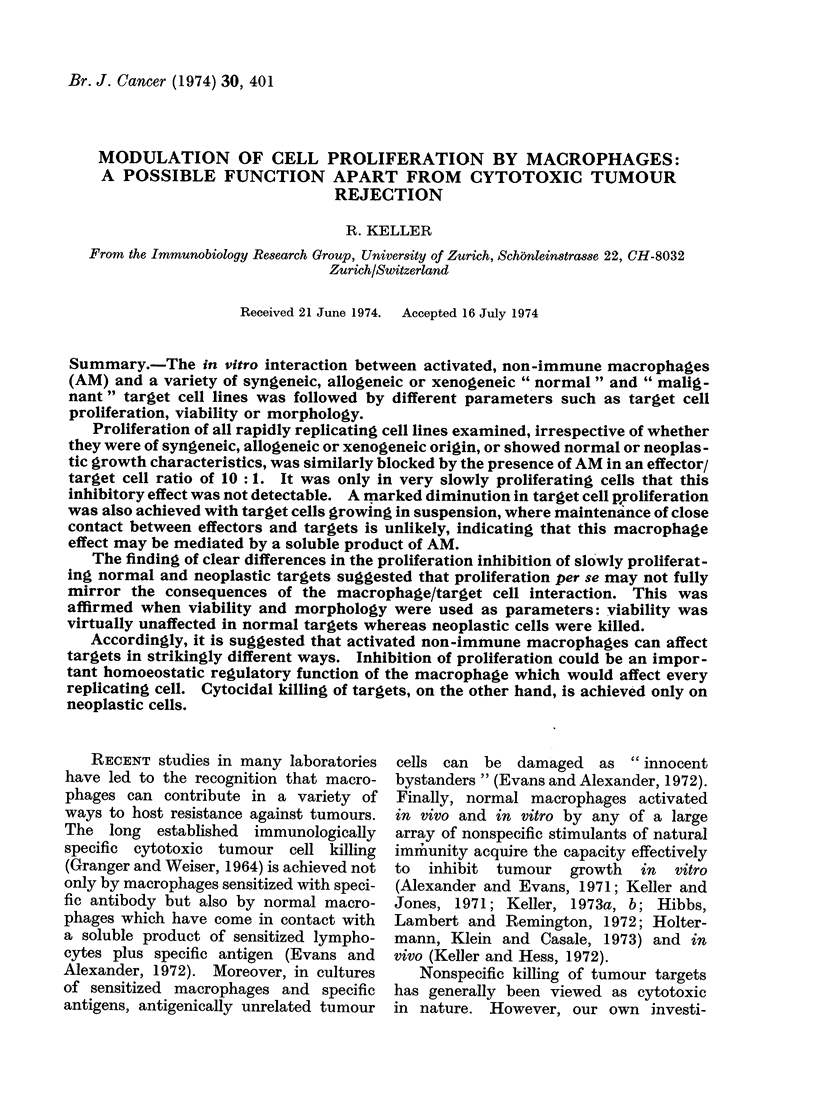

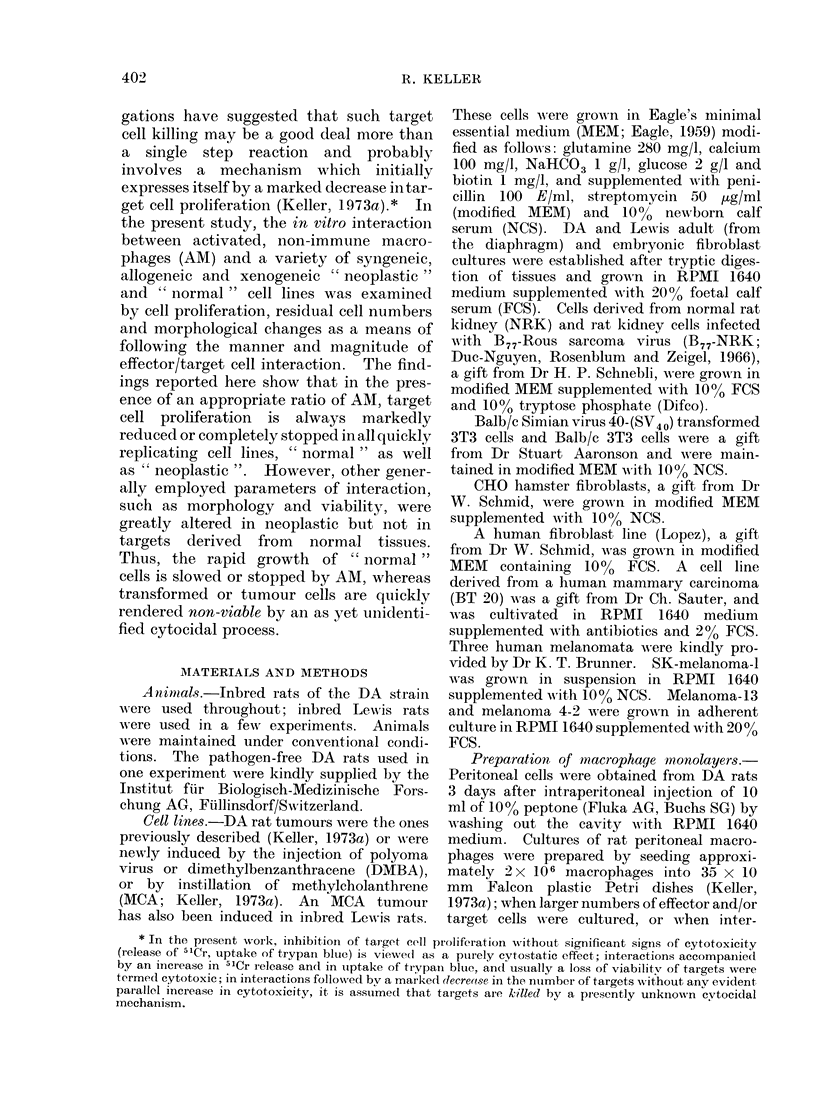

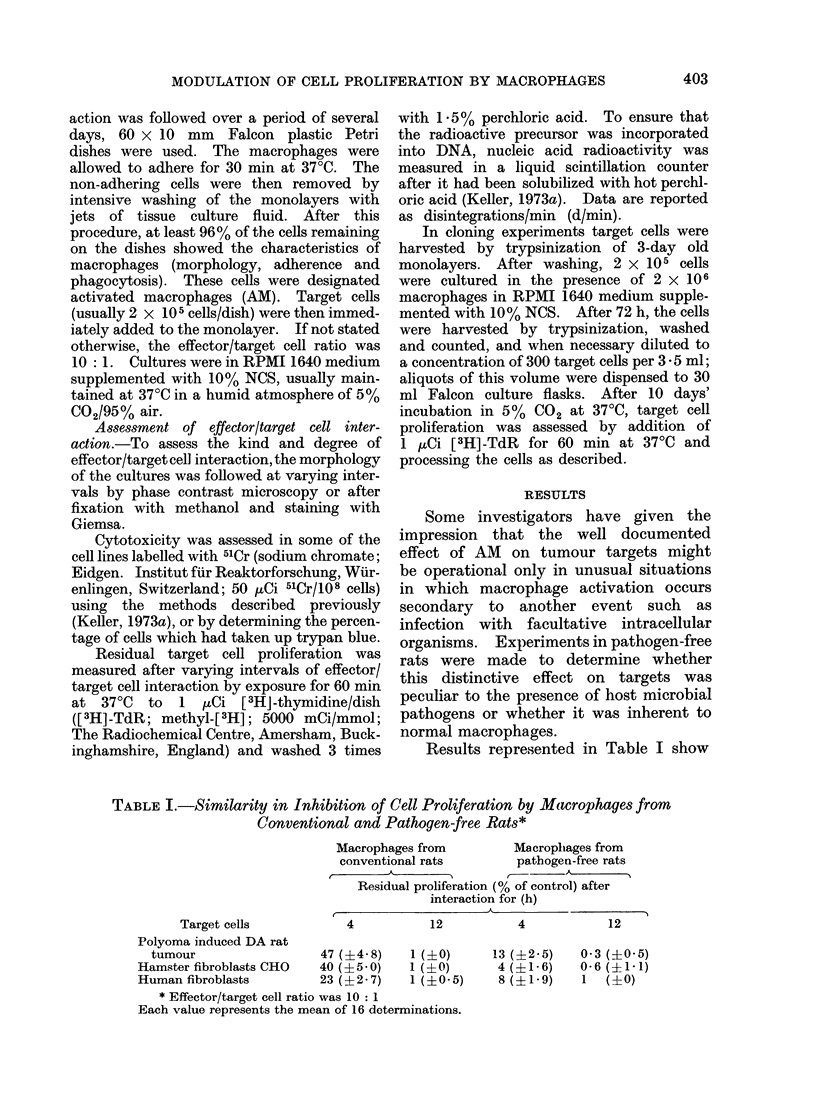

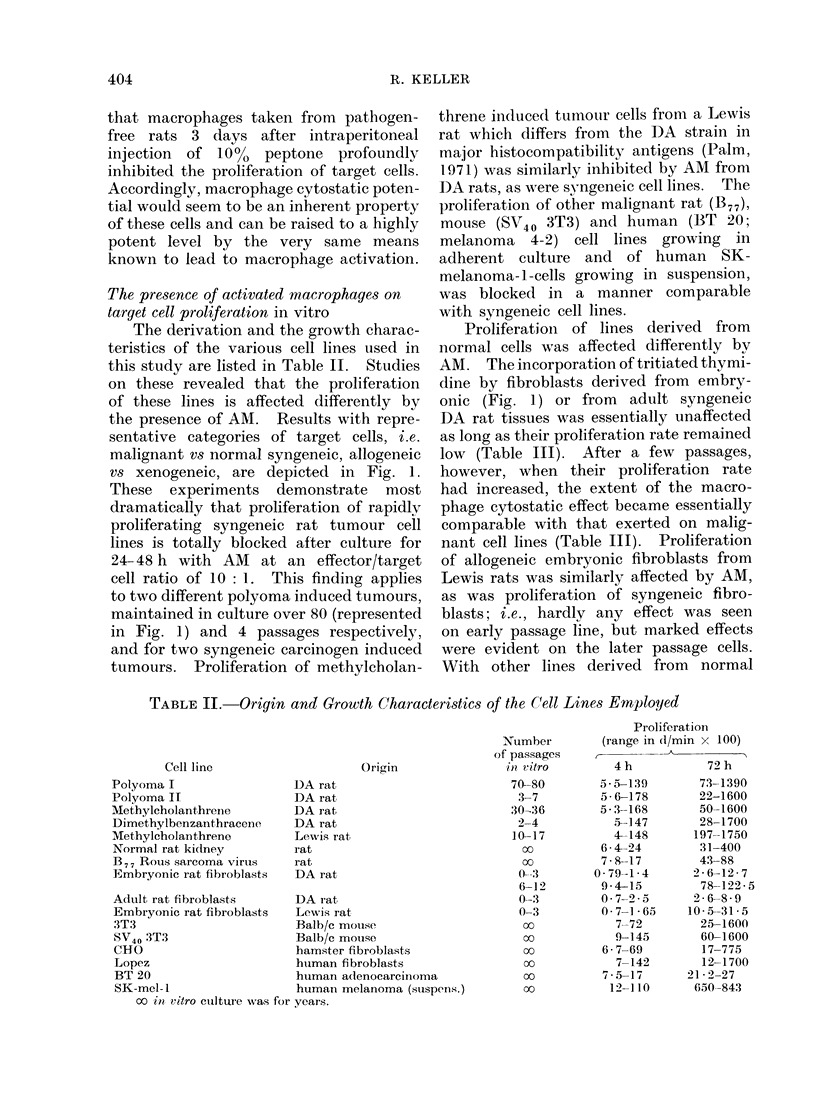

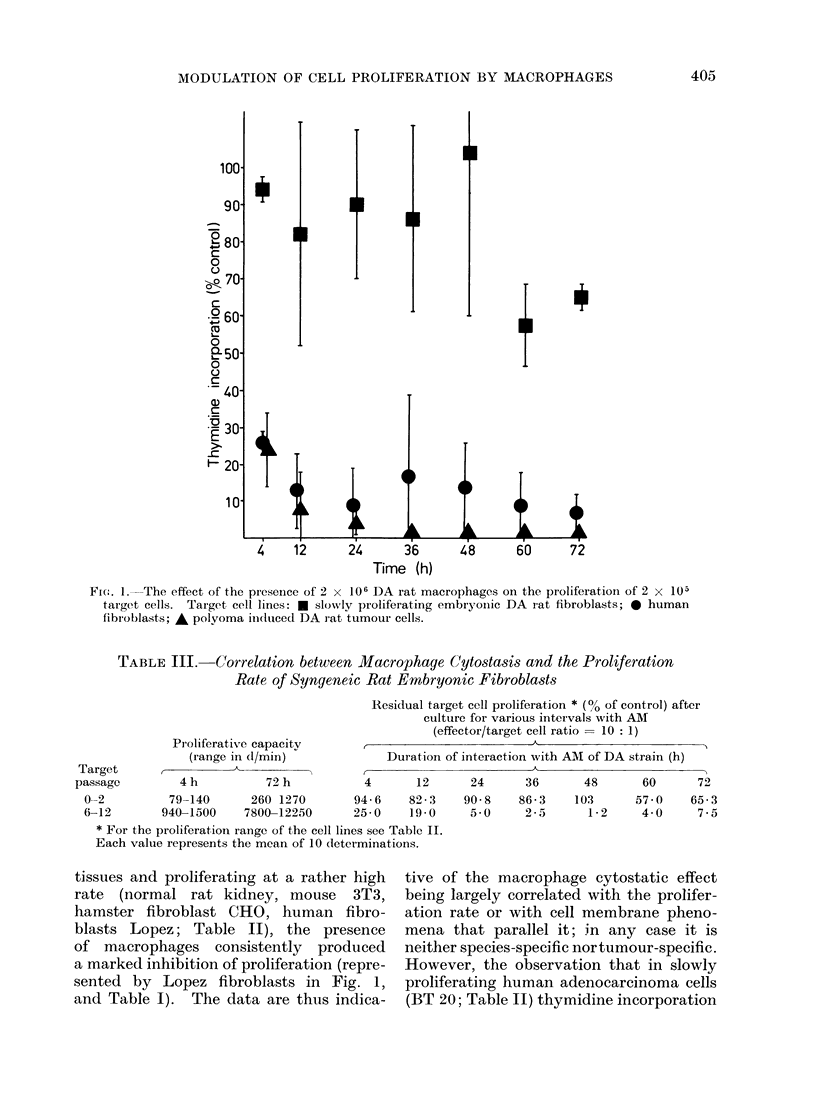

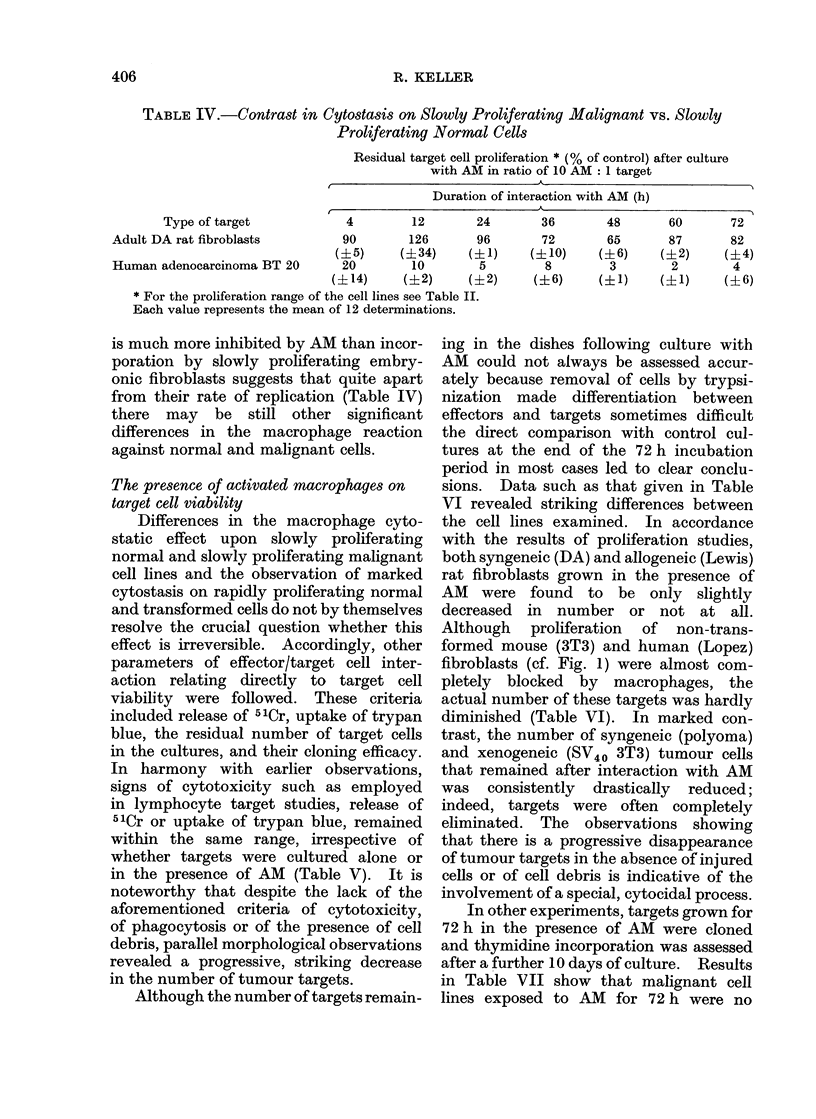

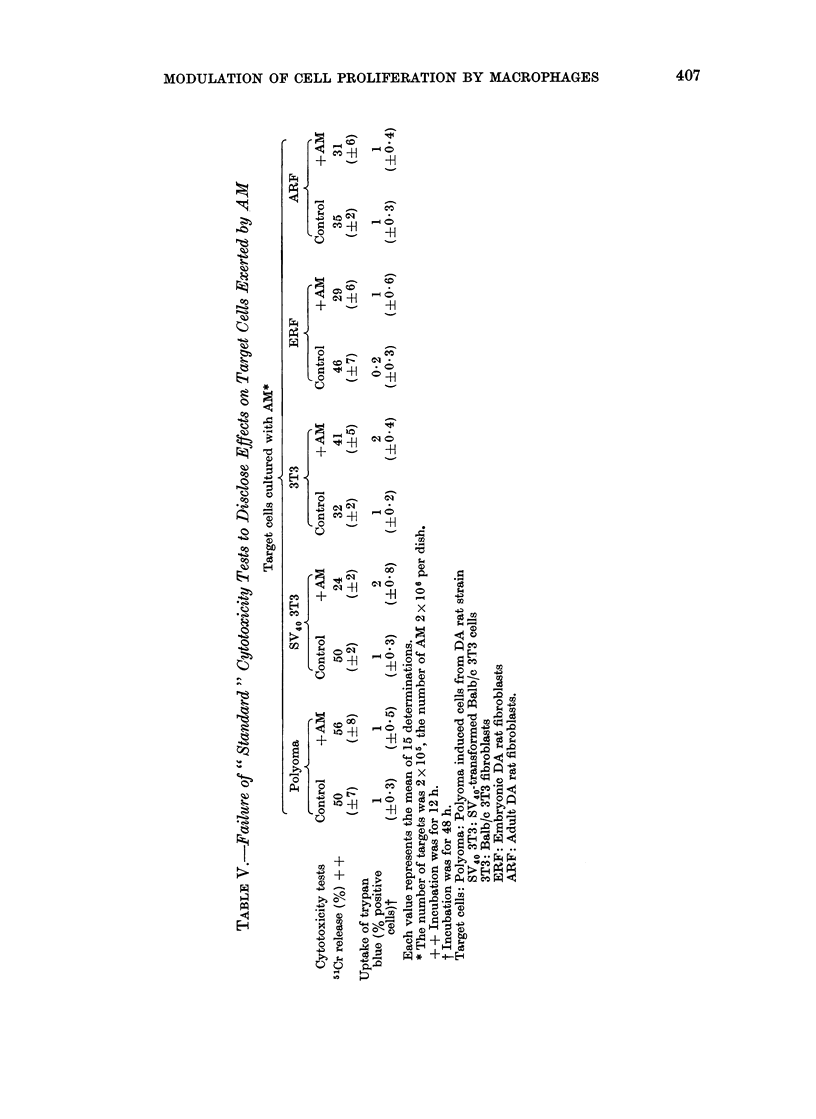

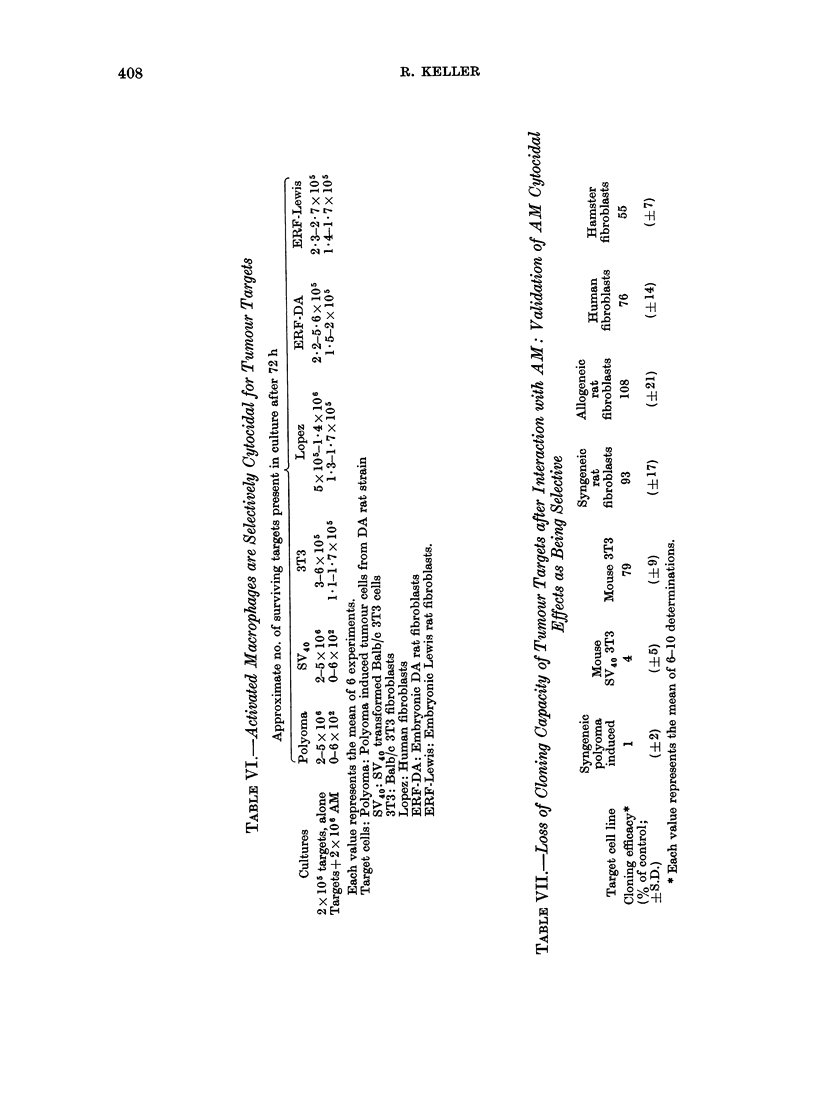

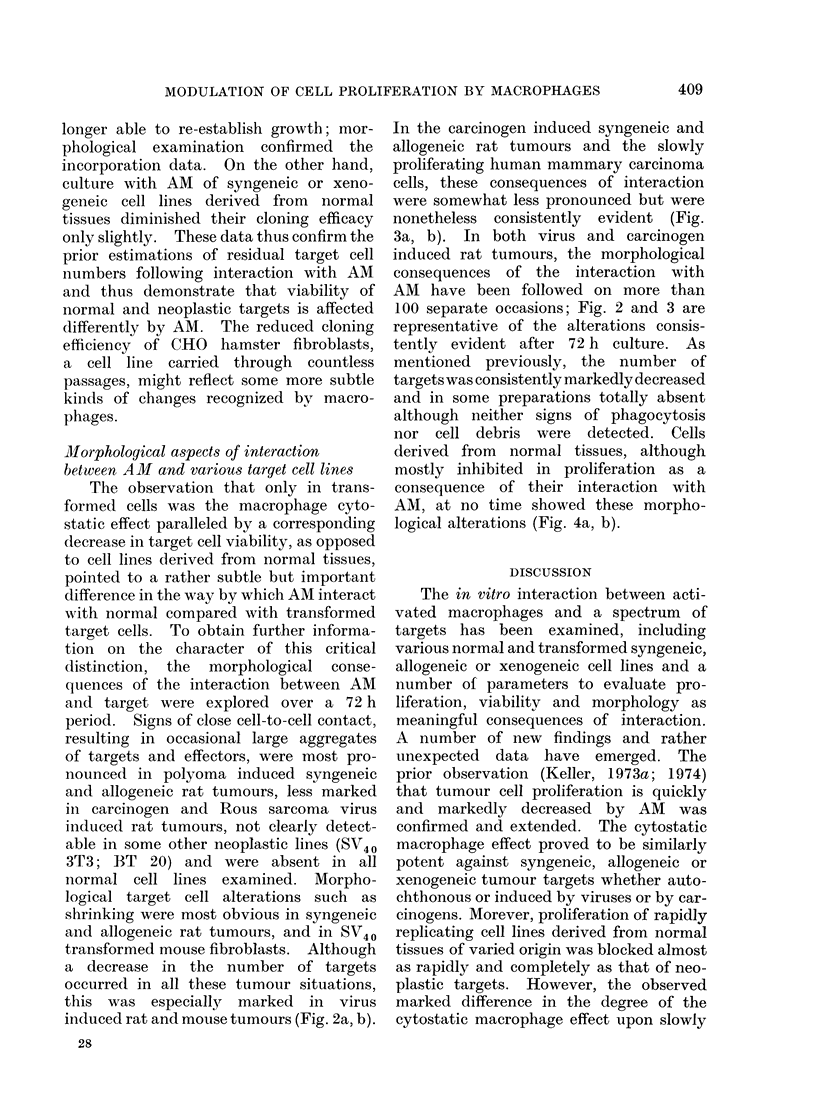

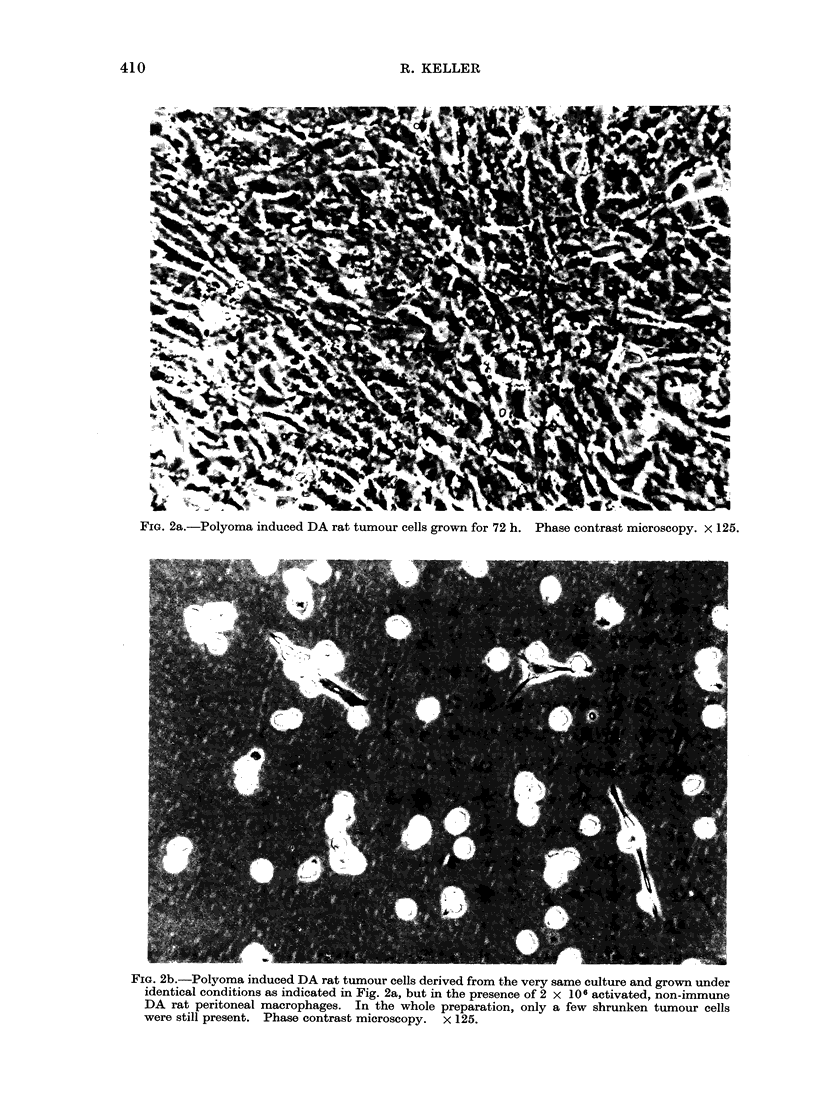

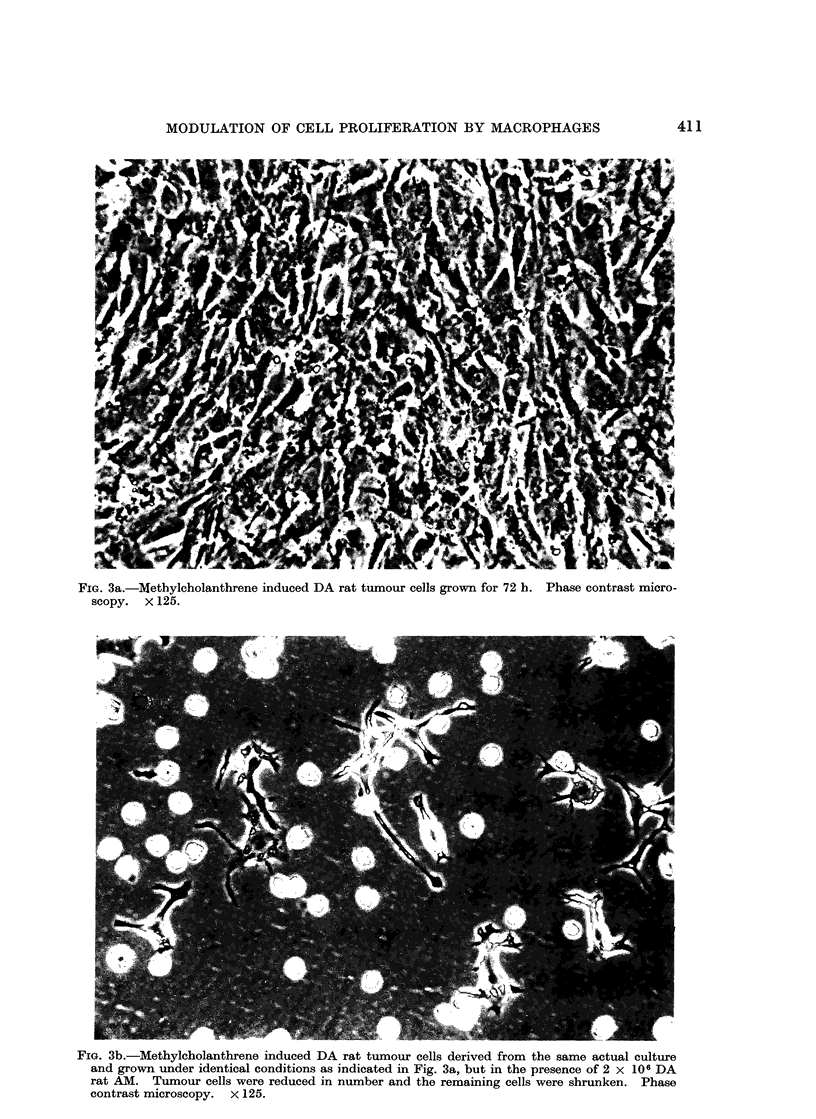

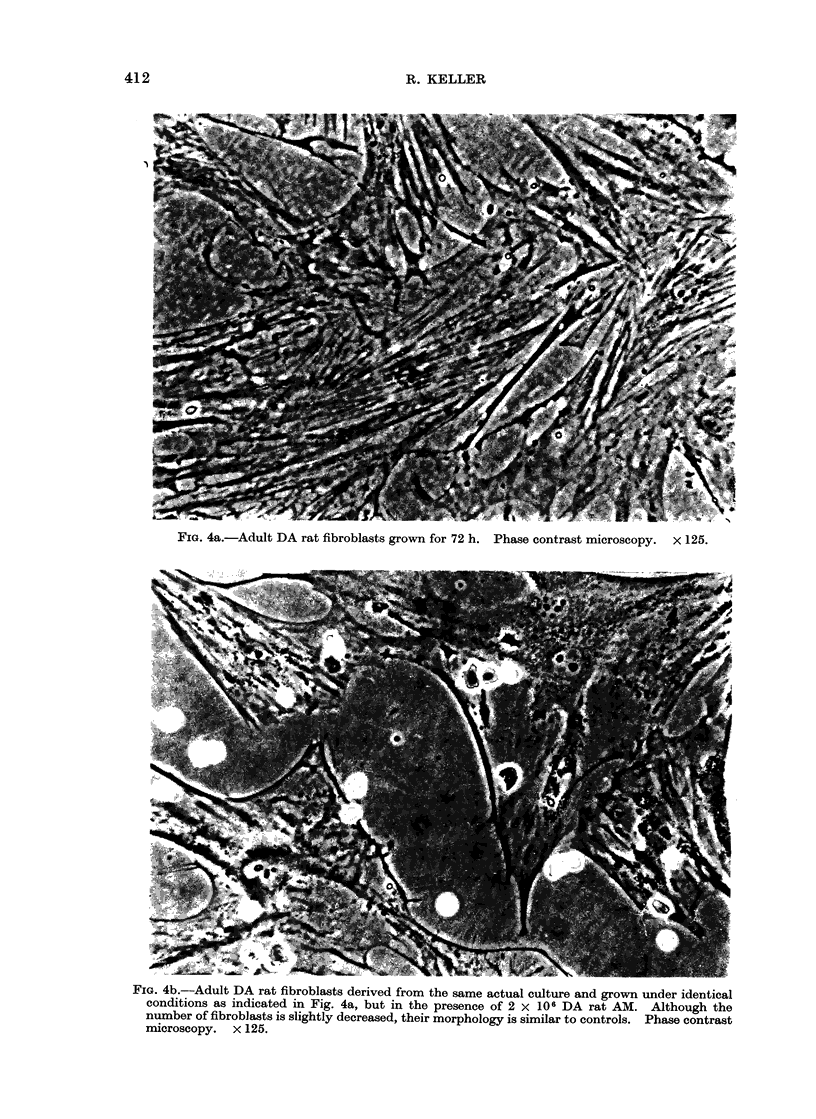

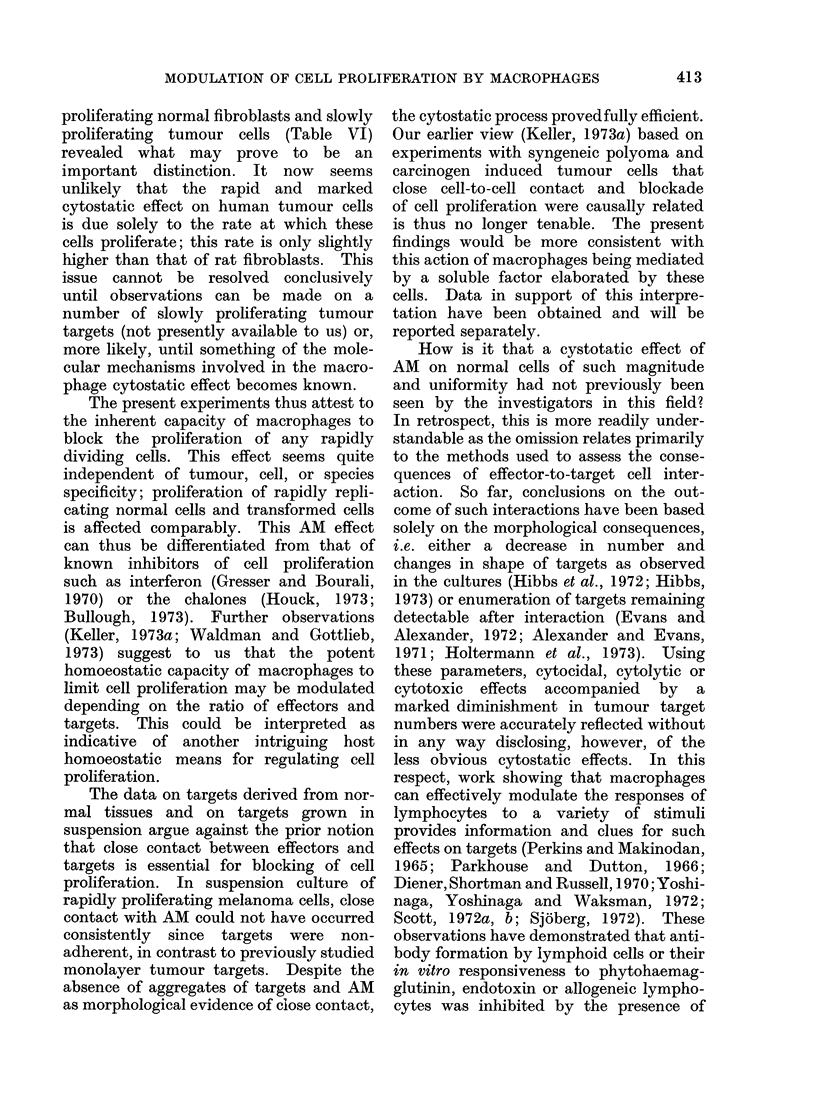

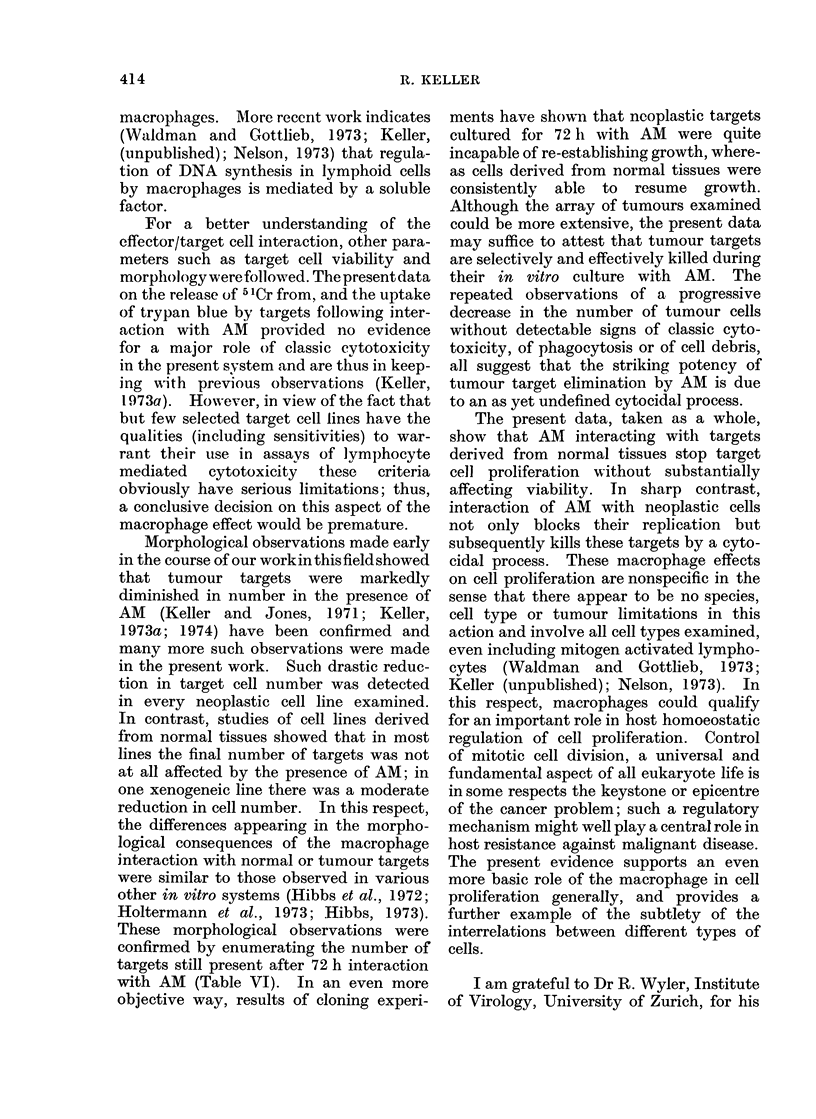

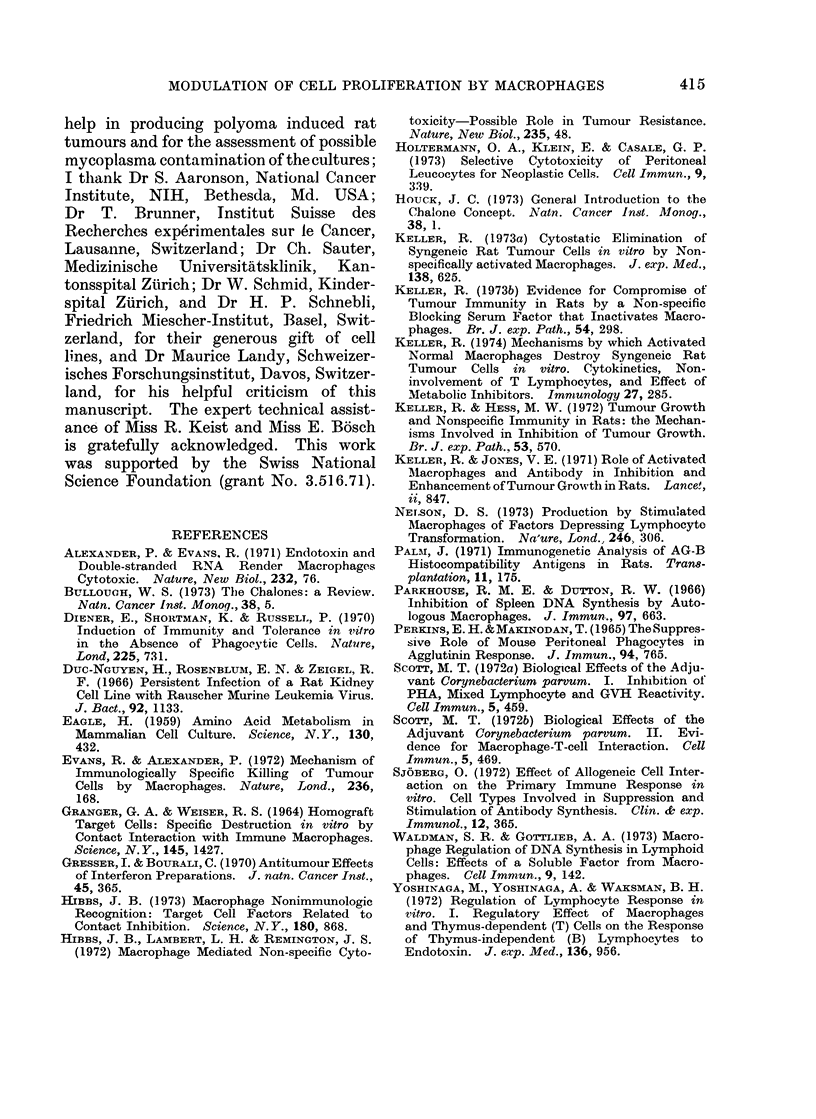


## References

[OCR_01340] Alexander P., Evans R. (1971). Endotoxin and double stranded RNA render macrophages cytotoxic.. Nat New Biol.

[OCR_01349] Diener E., Shortman K., Russell P. (1970). Induction of immunity and tolerance in vitro in the absence of phagocytic cells.. Nature.

[OCR_01361] EAGLE H. (1959). Amino acid metabolism in mammalian cell cultures.. Science.

[OCR_01366] Evans R., Alexander P. (1972). Mechanism of immunologically specific killing of tumour cells by macrophages.. Nature.

[OCR_01372] GRANGER G. A., WEISER R. S. (1964). HOMOGRAFT TARGET CELLS: SPECIFIC DESTRUCTION IN VITRO BY CONTACT INTERACTION WITH IMMUNE MACROPHAGES.. Science.

[OCR_01378] Gresser I., Bourali C. (1970). Antitumor effects of interferon preparations in mice.. J Natl Cancer Inst.

[OCR_01388] Hibbs J. B., Lambert L. H., Remington J. S. (1972). Possible role of macrophage mediated nonspecific cytotoxicity in tumour resistance.. Nat New Biol.

[OCR_01395] Holtermann O. A., Klein E., Casale G. P. (1973). Selective cytotoxicity of peritoneal leucocytes for neoplastic cells.. Cell Immunol.

[OCR_01355] Huu Duc-Nguyen, Rosenblum E. N., Zeigel R. F. (1966). Persistent infection of a rat kidney cell line with Rauscher murine leukemia virus.. J Bacteriol.

[OCR_01406] Keller R. (1973). Cytostatic elimination of syngeneic rat tumor cells in vitro by nonspecifically activated macrophages.. J Exp Med.

[OCR_01412] Keller R. (1973). Evidence for compromise of tumour immunity in rats by a non-specific blocking serum factor that inactivates macrophages.. Br J Exp Pathol.

[OCR_01427] Keller R., Hess M. W. (1972). Tumour growth and non-specific immunity in rats: the mechanisms involved in inhibition of tumour growth.. Br J Exp Pathol.

[OCR_01431] Keller R., Jones V. E. (1971). Role of activated macrophages and antibody in inhibition and enhancement of tumour growth in rats.. Lancet.

[OCR_01418] Keller R. (1974). Mechanisms by which activated normal macrophages destroy syngeneic rat tumour cells in vitro. Cytokinetics, non-involvement of T lymphocytes, and effect of metabolic inhibitors.. Immunology.

[OCR_01442] Palm J. (1971). Immunogenetic analysis of Ag-B histocompatibility antigens in rats.. Transplantation.

[OCR_01447] Parkhouse R. M., Dutton R. W. (1966). Inhibition of spleen cell DNA synthesis by autologous macrophages.. J Immunol.

[OCR_01457] Scott M. T. (1972). Biological effects of the adjuvant Corynebacterium parvum. I. Inhibition of PHA, mixed lymphocyte and GVH reactivity.. Cell Immunol.

[OCR_01476] Waldman S. R., Gottlieb A. A. (1973). Macrophage regulation of DNA synthesis in lymphoid cells: effects of a soluble factor from macrophages.. Cell Immunol.

[OCR_01482] Yoshinaga M., Yoshinaga A., Waksman B. H. (1972). Regulation of lymphocyte responses in vitro. I. Regulatory effect of macrophages and thymus-dependent (T) cells on the response of thymus-independent (B) lymphocytes to endotoxin.. J Exp Med.

